# Data on the determination of human epidermis integrity in skin permeation experiments by electrical resistance

**DOI:** 10.1016/j.dib.2018.10.098

**Published:** 2018-10-26

**Authors:** Umberto M. Musazzi, Antonella Casiraghi, Silvia Franzé, Francesco Cilurzo, Paola Minghetti

**Affiliations:** Department of Pharmaceutical Sciences, Università degli Studi di Milano, Via G. Colombo 71, 20133 Milano, Italy

## Abstract

The data presented in this article are related to the research article entitled “Design of in vitro skin permeation studies according to the EMA Guideline on quality of transdermal patches” (https://doi.org/10.1016/j.ejps.2018.09.014) (Cilurzo et al., 2018) [1].

In vitro permeation studies are generally carried out by Franz’s diffusion cell method using human epidermis as a membrane (Franz, 1975) [2]. The evaluation of membrane integrity is mandatory to assure the quality of the experiments. However, the methods used for this determination are different and the results are strictly dependent on the operative conditions. The article reports the electrical resistance values of human epidermis samples and in vitro skin permeability data of caffeine and benzoic acid. The data are used to establish a cut-off suitable for checking the skin integrity. This information may be useful to enable critical or extended analyses in order to contribute to the development of a compendial method.

**Specifications table**

**Table:** 

Subject area	*Pharmaceutical Sciences*
More specific subject area	*Evaluation of skin integrity for in vitro permeation tests*
Type of data	*Tables, Figures, Text file*
How data was acquired	*Electrical resistance measurement (*Agilent 4263B LCR Meter, Microlease, I)*, Franz’s diffusion cells (PermeGear, US)*
Data format	*Raw, Analyzed*
Experimental factors	*Electrical resistance values of human epidermis*
	*Permeation profiles of permeants through human epidermis*
Experimental features	*The cut-off of electrical resistance value for human epidermis used to discard damaged samples was set-up based on sodium dodecyl sulphate exposure and validated using caffeine and benzoic acid*
Data source location	*Milan, Italy*
Data accessibility	*The data is available within this article*
Related research article	[1]*Cilurzo et al. Design of in vitro skin permeation studies according to the EMA Guideline on quality of transdermal patches. Eur. J. Pharm Sci. 2018 125:86-92*

**Value of the data**

• The data permitted the definition of a cut-off (25 kΩ cm^2^) for electrical resistance measurements (voltage at 100 mV and frequency at 100 Hz) to select the human epidermis samples with acceptable permeability characteristics for the *in vitro* permeation studies using Franz’s diffusion cells.
• The data set can be useful to develop an inter-laboratory method based on electrical resistance to assess the epidermis integrity.
• The raw data on caffeine and benzoic acid permeation flux enriched the literature on the possible dependence of inter-donor variability from the physicochemical features of the permeant and electrical properties of the membrane.

## 1 Data

The determination of membrane electrical resistance (*R*) is an easy and fast method to verify the integrity of human epidermis samples used in in vitro permeation tests [1], [3].

**Fig. 1:**
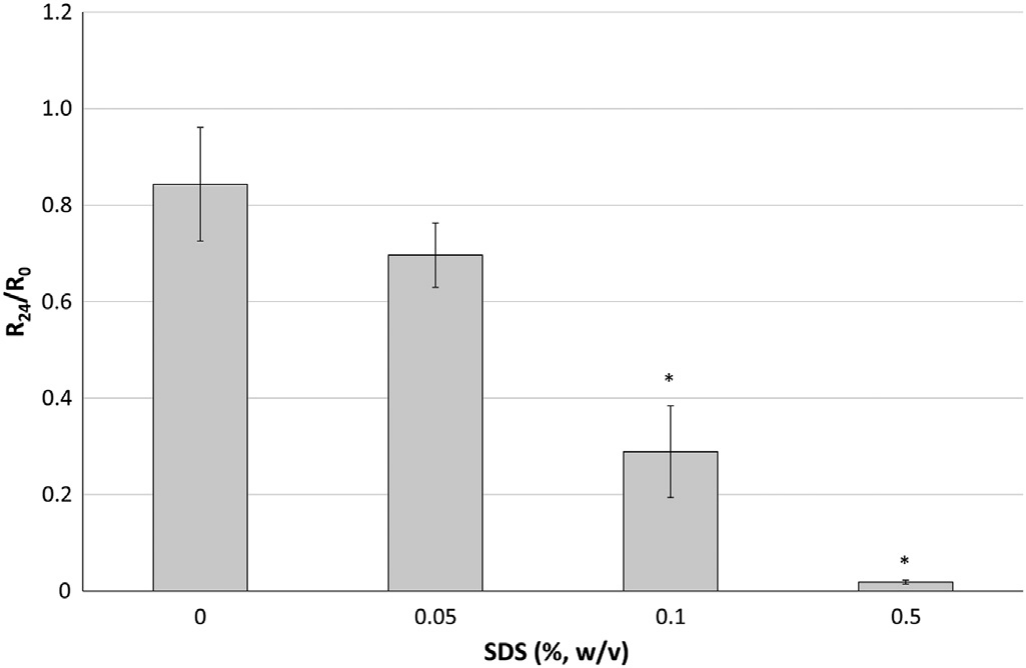
*R*_24_/*R*_0_ calculated after 24-h incubation of human epidermis with SDS solution (0.05 % - 0.5 % w/v) and purified water as control (mean ± dv.st; *n* = 3). * *p*-value < 0.05 in comparison to control.

The resistance values (*R*_0_) of epidermis samples obtained from three human donors ranged from 25.3 to 56.0 kΩcm^2^, immediately after being mounted on Franz’s diffusion cells. As shown in Fig. 1, when purified water was added in the donor compartment a slight decrease of *R*-values was observed after 24 h (*R*_24_= 35.6 ± 3.4 kΩcm^2^), due to the membrane hydration. On the other side, the exposure of epidermis to solutions of sodium dodecyl sulfate (SDS) determined the reduction of *R*-value, which is dependent on the surfactant concentration and the exposure time. Indeed, the *R*-value of human epidermis exposed to 0.5 % w/v SDS solution drastically dropped off to 6.9 ± 4.3 kΩcm^2^ (*R*_6_/*R*_0_ < 0.1) and to 0.8 ± 0.2 kΩcm^2^ (*R*_24_/*R*_0_ < 0.01, Fig. 1) after 6 h and after 24 h, respectively. The 0.1% w/v SDS halved the electrical resistance to 17.8 ± 5.3 kΩcm^2^ after 24 h. Instead, the R_24_-values after the treatment with a 0.05% w/v SDS solution (31.5 ± 7.1 kΩcm^2^) were not statistically different from the untreated epidermis samples. Furthermore, a direct correlation between the surfactant concentration and *R*-values was observed (*R*^2^: 0.81). Considering that it is well-known that SDS (≥ 0.1 % w/w) alters the integrity of human epidermis by disrupting the inner structure of the *stratum corneum* [4], these preliminary results suggested that 20–25 kΩcm^2^ can be used as cut-off for excluding damaged membranes. To confirm this cut-off, the impact of *R*-value on the permeation of 1 % w/v caffeine or 0.3 % w/v benzoic acid solutions through human epidermis was investigated. The *R*-values of the epidermis samples and the calculated *permeation fluxes (J)* of benzoic acid and caffeine are reported in Table 1 and Table 2, respectively. These model drugs were selected for their different physicochemical properties: the caffeine is a model of hydrophilic drugs, whereas benzoic acid a model of lipophilic drugs. In both cases, *J*-values increased for low *R*-values. In particular, using the Dixon’s *Q* test (*Q* = 95%), the *J*-values of benzoic acid can be considered outliers when the *R*-values are lower than 15 kΩcm^2^. Due to the well-known high inter-donor variability of caffeine, the same statistical approach cannot be applied to caffeine dataset since Dixon’s *Q* test excluded as outliers the data that had to be accepted on the bases of literature [5], [6]. Nevertheless, the caffeine *J* through the epidermis samples with an *R*-value lower than 20 kΩcm^2^ resulted almost 100-fold higher than those obtained with epidermis samples having an electrical resistance equal or higher than 25 kΩcm^2^.

**Table 1:** Electrical resistance data and permeation fluxes of benzoic acid (*J*_benz_) obtained by in vitro permeation studies. In bold the samples characterized by a low *R*-value.

**Donor ID**	***R* (kΩcm**^**2**^**)**	***J***_**benz**_**(µg/cm**^**2**^** h)**
1	20.16	52.86
1	19.59	72.56
2	22.81	62.44
2	47.07	50.78
**3**	**5.98**	**151.57**
4	23.02	65.93
5	22.81	58.30
5	17.68	64.72
6	32.23	42.90
6	33.98	38.89
**7**	**10.94**	**154.18**
7	19.59	43.43
**7**	**12.53**	**145.25**
9	31.42	57.77
11	44.09	46.57
11	23.02	54.77
12	31.42	66.34

**Table 2:** Electrical resistance data and permeation fluxes of caffeine (*J*_caff_) obtained by in vitro permeation studies. In bold the samples characterized by a low *R*-value.

**Donor ID**	***R* (kΩcm**^**2**^**)**	***J***_**caff**_**(µg/cm**^**2**^** h)**
**1**	**10.24**	**20.11**
**1**	**19.53**	**24.13**
2	29.14	1.35
3	36.30	0.83
3	40.99	0.23
4	34.85	0.22
4	29.68	0.35
**5**	**12.78**	**10.43**
**5**	**19.53**	**18.42**
8	33.68	0.83
8	36.25	1.45
9	25.36	0.40
10	28.87	0.40
10	56.55	0.55
11	38.22	0.85
12	33.39	0.53
12	47.51	0.50

On the bases of the actual dataset, all epidermis samples with an electrical resistance (measured with Agilent 4263B LCR Meter and setting the voltage at 100 mV and frequency at 100 Hz) lower than 25 kΩcm^2^ have to be discarded.

## 2 Experimental design, materials, and methods

### 2.1 Electrical resistance measurement after SDS exposure

The human epidermis samples were prepared following an internal standard procedure, as described by Casiraghi et al. [7]. The human epidermis samples were mounted on Franz’s diffusion cells [2], whose receptor compartments were filled with 0.9 % w/v saline solution. At the beginning of the experiment *R*-value (*R*_0_) was measured by the instrument Agilent 4263B LCR Meter (Microlease, Italy), setting the voltage at 100 mV and the frequency at 100 Hz. The analyses were carried out filling the donor compartment with 0.5 mL 0.9 % w/v saline solution. Then, the donor solution was withdrawn and replaced with 0.5 mL of SDS solution at different concentrations, namely 0.05 %, 0.1 % and 0.5 % w/v. The temperature of epidermis surface was kept at 32 ± 1 °C throughout the experiment. At different time, each donor compartment was washed twice with 0.9 % w/v saline solutions and the *R*-value (*R*_i_) was measured and compared to the initial values (*R*_0_). Purified water was used as a reference.

### 2.2 In vitro permeation of benzoic acid and caffeine through human epidermis

Thirty-four human epidermis samples obtained from different donors (Donor ID: 1-12) were mounted on Franz’s diffusion cells, whose receptor compartments were filled with 0.9 % w/v saline solution. At the beginning of the experiment *R*-value was measured by the instrument Agilent 4263B LCR Meter (Microlease, Italy), according to the experimental conditions reported above. Then, 0.5 mL of 1 % caffeine or 0.3 % benzoic acid solutions were added in the donor compartment. The in vitro permeation studies were performed using the same experimental protocol reported elsewhere [7]. The results were expressed as the average of parallel experiments performed in triplicate. The maximum flux (*J*) was determined as the slope of the linear portion of the plot of the cumulative amount permeated through the skin per unit area versus time.
